# Melatonin Prevents Myeloperoxidase Heme Destruction and the Generation of Free Iron Mediated by Self-Generated Hypochlorous Acid

**DOI:** 10.1371/journal.pone.0120737

**Published:** 2015-04-02

**Authors:** Faten Shaeib, Sana N. Khan, Iyad Ali, Tohid Najafi, Dhiman Maitra, Ibrahim Abdulhamid, Ghassan M. Saed, Subramaniam Pennathur, Husam M. Abu-Soud

**Affiliations:** 1 Departments of Obstetrics and Gynecology, the C.S. Mott Center for Human Growth and Development, Wayne State University School of Medicine, Detroit, Michigan, United States of America; 2 Department of Biochemistry and Genetics, Faculty of Medicine, An-Najah National University, Nablus, Palestine; 3 Department of Biochemistry and Molecular Biology, Wayne State University School of Medicine, Detroit, Michigan, United States of America; 4 Children’s Hospital of Michigan, Detroit, Michigan; 5 Division of Nephrology, Department of Internal Medicine, University of Michigan Medical School, Ann Arbor, Michigan, United States of America; University of the Balearic Islands, SPAIN

## Abstract

Myeloperoxidase (MPO) generated hypochlorous acid (HOCl) formed during catalysis is able to destroy the MPO heme moiety through a feedback mechanism, resulting in the accumulation of free iron. Here we show that the presence of melatonin (MLT) can prevent HOCl-mediated MPO heme destruction using a combination of UV-visible photometry, hydrogen peroxide (H_2_O_2_)-specific electrode, and ferrozine assay techniques. High performance liquid chromatography (HPLC) analysis showed that MPO heme protection was at the expense of MLT oxidation. The full protection of the MPO heme requires the presence of a 1:2 MLT to H_2_O_2_ ratio. Melatonin prevents HOCl–mediated MPO heme destruction through multiple pathways. These include competition with chloride, the natural co-substrate; switching the MPO activity from a two electron oxidation to a one electron pathway causing the buildup of the inactive Compound II, and its subsequent decay to MPO-Fe(III) instead of generating HOCl; binding to MPO above the heme iron, thereby preventing the access of H_2_O_2_ to the catalytic site of the enzyme; and direct scavenging of HOCl. Collectively, in addition to acting as an antioxidant and MPO inhibitor, MLT can exert its protective effect by preventing the release of free iron mediated by self-generated HOCl. Our work may establish a direct mechanistic link by which MLT exerts its antioxidant protective effect in chronic inflammatory diseases with MPO elevation.

## Introduction

Melatonin (MLT) is naturally synthesized from the amino acid tryptophan in the pineal gland, but also produced by other non-endocrine organs (e.g., cerebellum, cerebral cortex, retina, skin, ovary, liver, pancreas, kidneys, and immune competent cells), and acts through two G-protein coupled receptors, MT_1_ and MT_2_ [1–4]. In humans, as in most vertebrates, MLT operates as a modulator of circadian rhythms, and displays an oscillatory pattern through its unique ability to function as a signal, which organisms use to synchronize their circadian systems [3, 5, 6]. Multiple studies have shown that when MLT is administered either exogenously in vivo or when added to cultured cells via regulation of cellular pathways [3, 7–11] MLT has the ability to scavenge a wide range of reactive oxygen species (ROS) through its distinct antioxidant and anti-inflammatory effects [3, 7–11].The effects and action mechanisms of MLT belong to or take part in many different cell types including inflammatory cells such as monocytes–macrophages, neutrophils, eosinophils, basophils, mast cells, and natural killer cells [10, 12]. Therefore, various doses of synthetic MLT supplements have been used to treat a variety of medical scenarios in which inflammation plays a role such as a weakened immune system due to stress, oxidative hemolysis of red blood cells, and cancer progression [13–15]. Recently, we have shown that MLT is a potent inhibitor of the inflammatory enzymes myeloperoxidase (MPO) and other related peroxidases (e.g. eosinophil peroxidase) [16–18].

Myeloperoxidase is a heme protein, present in the neutrophils, which utilizes chloride (Cl^-^) in the presence of H_2_O_2_ to generate HOCl [19, 20]. This process occurs through H_2_O_2_ reduction that leads to the formation of MPO Compound I (ferryl porphyrin π cation radical, Fe(IV) = O(+π•)), which oxidizes Cl^-^ to HOCl [21]. Myeloperoxidase compound I is also capable of oxidizing various organic and inorganic substrates by two successive 1e− transfers to generate compound II (MPO-Fe(IV) = O) and MPO-Fe(III), respectively. The rate limiting step in a typical peroxidase cycle is the reduction of compound II to MPO-Fe(III). Furthermore, physiological reductants such as superoxide, nitric oxide, MLT, and ascorbic acid are known to accelerate this process [17, 22–26]. Hypochlorous acid is a potent oxidant that is capable, under normal circumstances, of functioning as a powerful antimicrobial agent [19, 20]. However, under a number of pathological conditions such as inflammatory diseases, in which ROS production can become excessive, HOCl is capable of mediating tissue damage [19, 27]. Interestingly, many inflammatory disorders such as ovarian cancer and atherosclerosis, in which MPO/HOCl have been known to be elevated, are also associated with significant free iron accumulation [28–31]. Recently, we have highlighted the potential link between elevated HOCl and hemoprotein heme destruction, and subsequent generation of free iron [21, 32, 33]. Detailed mechanistic insight into how exogenously added or self-generated HOCl mediates the MPO heme moiety has recently been elucidated [32, 34]. Therefore, factors that influence rates of HOCl removal are of growing interest [20, 35–40]. Here, we examine the ability of MLT to prevent HOCl-mediated heme destruction and subsequent iron release. These findings may have therapeutic repercussions as they elucidate the mechanism behind the rationale for additional studies on MLT supplementation for patients with chronic inflammatory conditions in which MPO is elevated. Additionally, this work may open the door for the development of other treatment interventions in this patient population.

## Materials and Methods

### Materials

All the materials used were of the highest-grade purity and used without further purification. Sodium hypochlorite (NaOCl), H_2_O_2_, ammonium acetate (CH_3_COONH_3_), ferrozine, MLT, ascorbic acid, and dimethylformamide, were obtained from Sigma Aldrich (St. Louis, MO, USA).

### General Procedures

#### MPO purification

MPO was initially purified from detergent extracts of human leukocytes by sequential lectin affinity and gel filtration chromatography [41–43]. Trace levels of contaminating eosinophil peroxidase (EPO) were then removed by passage over a sulfopropyl Sephadex column [43]. Purity of isolated MPO was established by demonstrating a Reinheitszahl (RZ) value of greater than 0.85 (A_430_/A_280_), SDS−PAGE analysis with Coomassie Blue staining, and gel tetramethylbenzidine peroxidase staining to confirm no contaminating EPO activity. Enzyme concentration was determined spectrophotometrically utilizing extinction coefficients of 89,000 M^-1^ cm^-1^/heme of MPO [44].

#### H_2_O_2_-selective electrode measurements

Hydrogen peroxide measurements were carried out using an H_2_O_2_-selective electrode (Apollo 4000 free radical analyzer; World Precision Instruments, Sarasota, FL, USA). Experiments were performed at 25°C by immersing the electrode in 3 ml of 0.2 M sodium phosphate buffer, pH 7.0. Experiments were carried out under two different conditions: sequential additions of 10 μM H_2_O_2_ to a continuously stirred buffer solution supplemented with 40 nM MPO and 100 mM Cl^-^ in the absence and presence of 200 μM MLT during which the change of H_2_O_2_ concentration was continuously monitored.

#### Absorbance measurements

The absorbance spectra were recorded using a Cary 100 Bio UV–visible photometer at 25°C, pH 7.0. Experiments were performed in 1 ml phosphate buffer solution supplemented with MPO (1.0–1.5 μM), 100 mM Cl^-^, and incremental additions of 180 μM of H_2_O_2_ (20 μM; 2 μl) in the absence and presence of increasing MLT concentrations (0–200 μM). After each H_2_O_2_ addition, the reaction mixture was left for 10 minutes for reaction completion and absorbance spectra were then recorded from 300 to 700 nm.

#### Free iron analysis

Free iron release was measured colorimetrically using ferrozine, following a slight modification of a published method [45]. To 100 μl of the sample (MPO–HOCl reaction mixture), 100 μl of ascorbic acid (100 mM) was added. After 5 min of incubation at room temperature, 50 μl of ammonium acetate (16%) and the same volume of ferrozine (16 mM) were added to the mixture and mixed well. Subsequently, the reaction mixture was incubated for 5 min at room temperature and the absorbance was measured at 562 nm. A standard curve prepared using ammonium Fe(III) sulfate was used for the calculation of free iron concentration. Final concentrations of the additives were as follows: ascorbic acid, 33.33 μM; ammonium acetate, 5.3%; and ferrozine, 5.3 μM.

#### High Performance Liquid Chromatography (HPLC)

HPLC analyses were performed using a Shimadzu HPLC system equipped with an SCL-10A controller, LC-10 AD binary solvent delivery pumps, SIL-10 AD autosampler, SPD-M10 A diode array detector, and an RF-10 A XL fluorescence detector. An Alltech 5 μm particle size column was used with a 4.6 x 150 mm reverse phase octadecylsilica (C18). To monitor the chromatogram, the RF fluorescence detector was set at 321 nm for excitation and 465 nm for emission and the SPD diode array detector was set at 400 nm. HPLC grade solvents were prepared as follows: solvent A, 0.1% TFA in water and solvent B, 0.1% TFA in 80% acetonitrile. Solvent gradients were set as follows: 0–10 min 55–65% B, 10–14 min 65–90% B, followed by reducing solvent B composition to 55% within 14–24 min. The column elution was carried out at a flow rate of 0.8 ml/min with a linear gradient of solvents. After treatment of MLT with MPO in presence of H_2_O_2_ for 24 hours, the reaction mixture was filtered through an Amicon Ultra-15 centrifugal filter unit with Ultracel-10 membrane (from Millipore) 3-kDa cut-off by centrifuging at 14,000 rcf rate for 30 min at 4°C; then 50 μl of filtered sample was injected for analysis. At the end of the run the system was equilibrated with 45% solvent A; each sample was analyzed in triplicate.

#### N^1^-acetyl-N^2^-formyl-5-methoxykynuramine (AFMK) synthesis

The AFMK was synthesized following the method of Tan et al. [46] with slight modification. Briefly, 5 mg MLT were dissolved in 100 μL methanol and the reaction mixture was mixed with 500 μL H_2_O_2_ (30%). The formation of AFMK was followed by the increase in absorbance at 340 nm. AFMK was isolated and confirmed by HPLC analyses.

### Solution preparation

#### HOCl preparation

HOCl was prepared as previously described with some modifications [47]. Briefly, a stock solution of HOCl was prepared by adding 1 ml of NaOCl solution to 40 ml of 154 mM NaCl and the pH was adjusted to around 3 by adding HCl. The concentration of active total chlorine species in solution, expressed as [HOCl]_T_ (where [HOCl]_T_ = [HOCl] + [Cl_2_] + [Cl_3_^−^] + [OCl^−^]) in 154 mM NaCl, was determined by converting all the active chlorine species to OCl^−^ by adding a bolus of 40 μl of 5 M NaOH and measuring the concentration of OCl^−^. The concentration of OCl^−^ was determined spectrophotometrically at 292 nm (ε = 362 M^− 1^ cm^− 1^). As HOCl is unstable, the stock solution was prepared on a daily basis, stored on ice, and used within 1 hour of preparation. For further experimentation, dilutions were made from the stock solution using 200 mM phosphate buffer, pH 7, to give working solutions of lower HOCl concentrations.

#### Melatonin solution

A stock solution of MLT was dissolved in dimethylformamide (DMF) and then diluted to the required concentrations with phosphate buffer (pH = 7.00). The concentration of DMF in all MLT solutions was less than 1% and did not interfere with MPO activity.

## Results

Melatonin prevents MPO inactivation by HOCl generated during MPO steady-state catalysis: The ability of MLT to prevent HOCl damage to MPO catalytic activity was determined by two methods. The first involved the use of an H_2_O_2_-selective electrode, which measured the first step in the MPO catalytic cycle in which H_2_O_2_ is consumed by MPO. The second method measured HOCl–mediated MPO heme destruction utilizing UV-Visible and free iron release using ferrozine assay.

**Fig 1:**
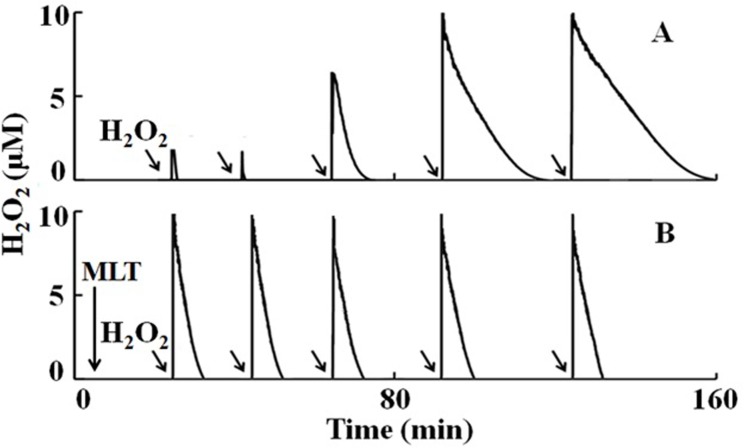
Melatonin inhibits MPO chlorination activity and prevents MPO heme destruction and iron release mediated by MPO self-generated HOCl. (A) A typical recording by an H_2_O_2_-selective electrode demonstrating the dramatic MPO feedback inhibition mediated by self-generated HOCl after addition of equal amounts of H_2_O_2_ (10 μM, 1–2 μl in 3 ml reaction mixture) five consecutive times (denoted by the arrows) to a continuously stirred phosphate buffer (200 mM, pH 7.4) containing 40 nM MPO and 100 mM Cl^−^, at 25°C. (B) Similar experiment was repeated in the presence of MLT (100 μM), showing a significant protection of peroxidation activity of MPO. Under these circumstances, MLT inhibits the chlorinating activity of MPO and no heme destructions have been observed [17]. The data shown are representative of three independent experiments.

The H_2_O_2_-selective electrode measurements revealed that addition of an aliquot of H_2_O_2_ (10 μM; 3.5 μL) to the continuously stirred reaction solution supplemented with 40 nM MPO and 100 mM Cl^-^ demonstrated an instant consumption of H_2_O_2_, as previously reported [26, 35, 36]. Subsequent multiple additions of the same amount of H_2_O_2_ to the MPO/Cl^-^ solution mixture caused MPO inhibition, as judged by the accumulation of H_2_O_2_ (amplitude of H_2_O_2_ signal) and a slower rate of its consumption (longer duration) (Fig. 1A). Under these circumstances, self-generated HOCl inhibited MPO through a mechanism that involves heme destruction, precluding the enzyme from functioning at maximum activity (80–90% inhibition) [34] (Fig. 1A). We used 40 nM MPO as the catalytic concentration. The pathophysiological effect of MPO was shown at concentrations of 5 nM [48]. To examine whether MLT could prevent the feedback heme destruction mediated by HOCl, an identical experiment was repeated in the presence of a saturating amount of MLT. Addition of H_2_O_2_ (10 μM) to a continuously stirred buffer solution supplemented with 40 nM MPO, 100 mM Cl^-^, and 200 μM MLT caused a much slower rate of H_2_O_2_ consumption compared to the control solution (Fig. 1B), which indicated that MLT inhibited the MPO catalytic activity [49]. The repeated addition of the same amounts of H_2_O_2_ to the MPO/Cl^-^/MLT reaction mixture showed that the degree of MPO inhibition remained the same for all five of the trials. Thus under these conditions, MLT protected the peroxidation activity of MPO, but inhibited the chlorinating activity of the enzyme by serving as 1 e^-^ substrate for both MPO compounds I and II [17].

**Fig 2:**
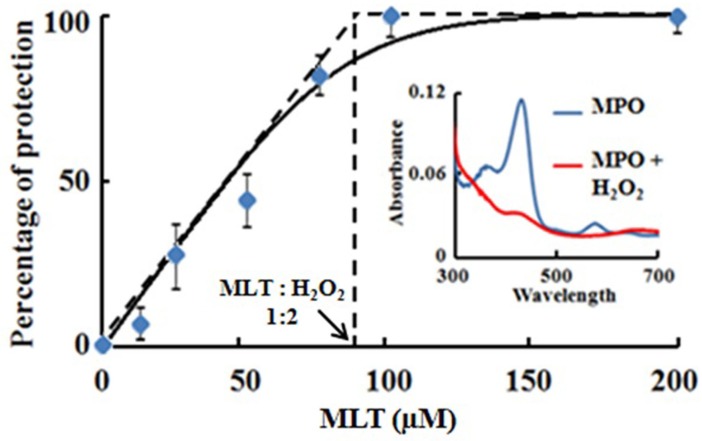
Melatonin prevents MPO heme destruction mediated by self-generated HOCl during steady state catalysis. Fixed amount of MPO (1 μM) was incubated with fixed amount of Cl^-^ (100 mM) and increasing concentration of MLT (12–200 μM), and the reaction mixtures were incrementally received fixed amount of H_2_O_2_ (20 μM, total concentration of 180 μM). After reaction completion, the spectra of the reaction mixtures were scanned from 300–700 nm.

We next performed UV-visible photometry to correlate the degree of catalytic inhibition with HOCl-mediated heme destruction. As shown in the Fig. 2 inset; blue trace, MPO-Fe(III) as isolated displays a Soret absorbance peak centered at 430 nm, with three additional peaks at 573, 630, and 694 nm. Since the addition of a high molar ratio of H_2_O_2_ to MPO causes the conversion of MPO to Compound (III) (MPO-FeII-O_2_ complex) [50], the oxidation of the MPO heme moiety mediated by self-generated HOCl was monitored by sequential addition of H_2_O_2_ (20 μM; 3 μl) (180 μM H_2_O_2_ total) to the MPO-Fe(III)/Cl^−^ mixture. With each incremental addition of H_2_O_2_, there was a proportional decrease in the MPO Soret peak, indicating that HOCl-mediated MPO feedback inhibition is associated with MPO heme destruction. After the last addition of H_2_O_2_ (180 μM total) solution to enzyme mixture, the spectrum recording showed a flattening in the Soret peak at 430 nm indicating MPO heme destruction (Fig. 2 inset; red trace). This flattening in the Soret peak region occurred solely in the presence of Cl^−^, signifying HOCl to be the major cause of MPO heme destruction. To confirm that MLT prevents HOCl-mediated MPO heme destruction, a fixed amount of MPO/Cl^−^ mixture was preincubated with increasing concentrations of MLT prior to incremental additions of H_2_O_2_ to the reaction mixture. Fig. 2 shows the percentage recovery of MPO heme content, measured at 430 nm after the last addition of the incremental H_2_O_2_ to the enzyme solution, as a function of MLT concentration. In the presence of a saturating amount of MLT (>100 μM), spectral analysis indicated no losses in the heme content. Under these conditions, the MPO-H_2_O_2_ system utilized MLT as a 1e^-^ substrate for the formation and subsequent decay of Compound II. The accumulation and stability of MPO Compound II (characterized by a Soret absorbance peak at 450 nm) during catalysis depended on the MLT concentration. In the presence of lower MLT levels, addition of limited amounts of H_2_O_2_ (10 μM) to the solution mixture caused immediate appearance of MPO Compound II, which then decayed to the ferric form in the next few seconds. In the presence of higher MLT concentrations (e.g. 100–400 μM), no significant change in absorbance was observed upon the addition of an H_2_O_2_ solution to the MPO mixture, indicating that the rate of MPO compound II decay exceeded the rate of formation, which was consistent with previous results [51]. In the presence of 50 μM MLT, only 50% recovery was noted in the MPO Soret absorbance peak of the total enzyme. As shown in Fig. 2, the full protection of the MPO heme contents required the presence of a ratio 1:2 MLT: H_2_O_2_. Collectively, our results showed that heme destruction did not occur in the presence of MLT, where MPO began reducing H_2_O_2_ without generating HOCl, indicating that self-generated HOCl is the major cause of MPO inactivation.

The percent recovery in MPO Soret peak (430 nm) plotted as a function of melatonin concentration. The full protection of the MPO heme contents required the presence of a ratio 1: 2 MLT: H_2_O_2_. The inset shows the absorbance spectra of MPO ferric form before (blue trace) and after the last incremental addition of H_2_O_2_ (red trace). The flattening in the MPO spectrum indicates MPO heme destruction. The data points are the average of three independent experiments.

**Fig 3:**
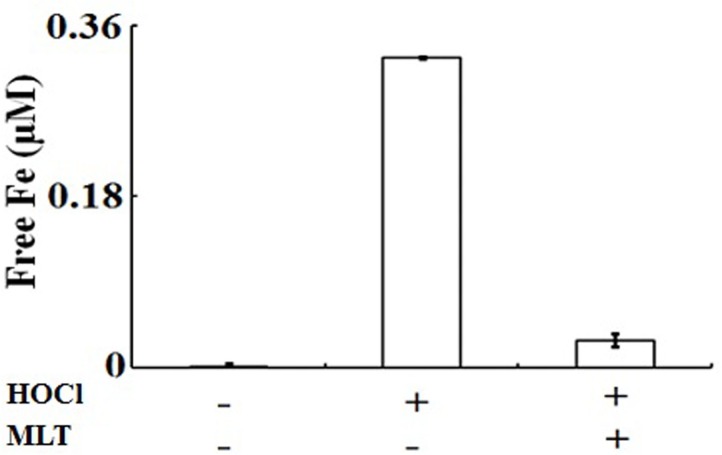
Melatonin prevents HOCl mediated MPO heme destruction and subsequent free iron release during MPO catalysis. MPO (1.2 μM) was incubated with 100 mM Cl^−^ in the absence and presence of 400 μM MLT followed by the addition of aliquots of H_2_O_2_ (in increments of 20 μM) to the reaction mixture. The free iron released was measured using ferrozine assay as detailed under Materials and methods. No free iron was detected before the addition of H_2_O_2_. The data are the averages of three independent experiments with the error bars representing the standard error of measurement.

To investigate how the flattening in the Soret absorbance peak at 430 nm in H_2_O_2_-treated samples is linked to MPO heme depletion and if MLT can prevent this finding, we studied the free iron release after H_2_O_2_ treatment in the absence and presence of saturating amounts of MLT. Compared to the free iron content of the untreated control, treatment with H_2_O_2_ led to a significant increase in free iron content (Fig. 3). Additionally, in the same figure, we noted around 25% free iron detection. This finding is likely secondary to the fact that not all iron was detached from the heme fragments, and therefore not able to be detected by the assay. The accumulation of free iron significantly decreased in the presence of saturating amounts of MLT, confirming the above spectrophotometric studies. Thus, MLT not only inhibits MPO catalytic activity, but also prevents heme destruction and subsequent free iron release mediated by self-generated HOCl.

**Fig 4:**
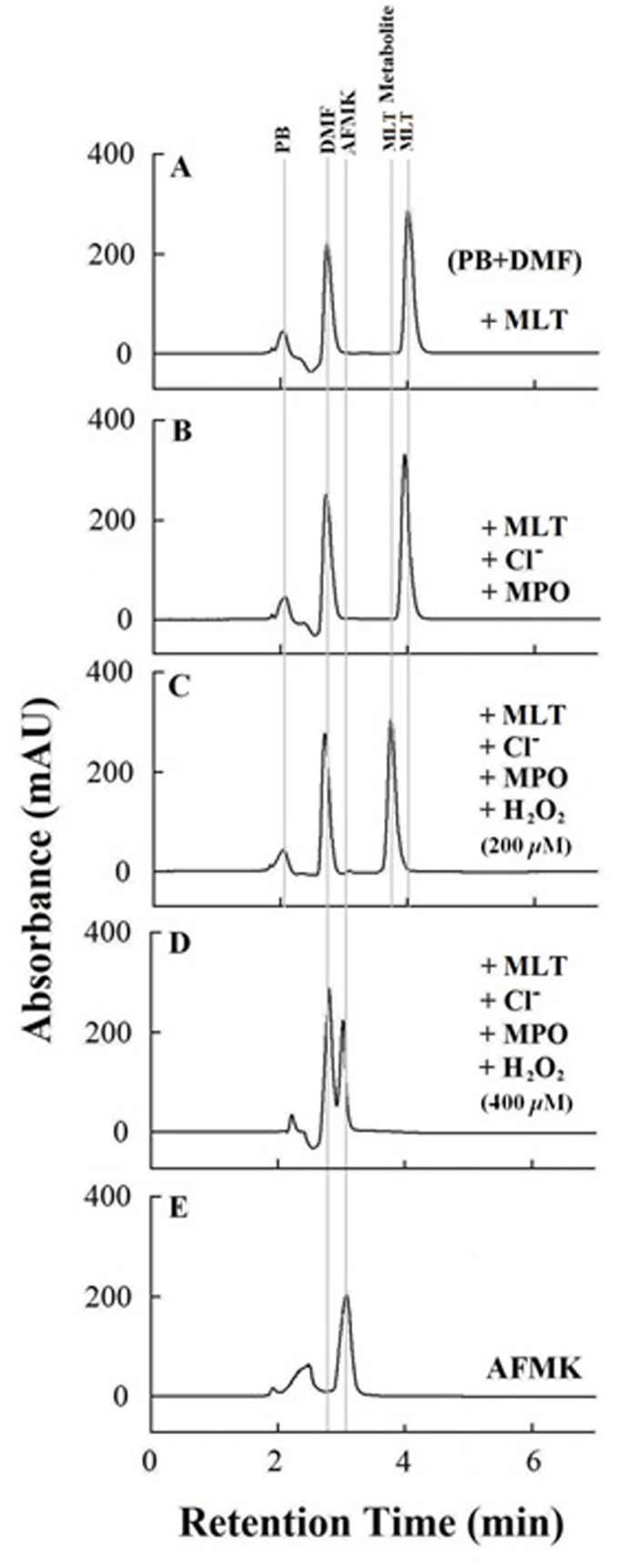
HPLC analysis shows MLT oxidation thereby preventing MPO heme destruction and generation of free iron. A) HPLC trace for MLT (elution time 3.98 min) dissolved in DMF (elution time 3.31 min) and phosphate buffer (elution time 2.48 min). B) Addition of MPO and Cl^-^ causes no significant change in MLT peak intensity and/or retention time. C) Addition of H_2_O_2_ (sequential addition of 20 μM, total 200 μM) results in a significant shift in MLT retention time elution time (3.71 min) as well as the appearance of a small peak around 3.57 min. D) Increasing levels of H_2_O_2_ (400 μM) resulted in the domination of the MLT metabolite eluted at 3.57 min showing the retention and absorbance properties of AFMK (elution time 3.57 min) as shown in panel (E).

The protection of MPO heme destruction mediated by self-generated HOCl occurred at the expense of melatonin oxidation:

Finally, HPLC analysis (anion exchange) was utilized to investigate in depth the mechanism by which MLT presence prevents MPO heme destruction mediated by self-generated HOCl. Using this method, we observed an accumulation of two major MLT metabolites when concentrations of MLT used were sufficient to produce dramatic effects on the rates of Compound II formation, duration, and decay. The population of these metabolites is varied and dependent on the H_2_O_2_ concentration used (Fig. 4). HPLC analyses were conducted under five different conditions: MLT alone; MPO (40 nM) pre-incubated with MLT (100 μM) alone; the solution mixture of MPO pre-incubated with MLT (100 μM) which received sequential additions of 20 μM H_2_O_2_ (to total either 200 or 400 μM H_2_O_2_); and finally AFMK alone. After reaction completion, the reaction mixtures were filtered to eliminate MPO and the supernatants were then injected into the HPLC system. Under our experimental conditions phosphate buffer and DMF were eluted at 2.48 and 3.31 min, respectively (Fig. 4). MLT alone was eluted at 3.95 min (Fig. 4A) while AFMK alone was eluted at 3.57 min (Fig. 4E), and both were identified by their characteristic spectra observed from the photodiode array detector at 222 and 236 nm, respectively. Pre-incubation of MLT (100 μM) with a catalytic amount of MPO (40 nM) and Cl^-^ (100 mM), in the absence of H_2_O_2_, fails to generate any detectable MLT metabolite (Fig. 4B). HPLC analysis also indicated that the relatively short incubation times of H_2_O_2_ at the different concentrations employed (200–400 μM) in the experiment for 2h has no effect on the MLT (100 μM) moiety (data not shown). However, long incubation of high concentration of H_2_O_2_ (30%) with MLT in the presence of methanol generates AFMK (Fig. 4E) [46].

However, incremental addition of H_2_O_2_ to the enzyme-MLT mixture resulted in MLT oxidation to a lower elution time indicating formation of MLT metabolites with lower hydrophobicity. The enzyme sample that was pre-incubated with 100 μM MLT and treated with 200 μM H_2_O_2_ (total) led to the production of two main MLT metabolites with elution times of 3.71 and 3.57 min with the first being the most abundant and attributed to the formation of hydroxyl melatonin metabolite (Fig. 3C). Although we provide no direct evidence for the hydroxyl melatonin formation, we do note corresponding 1e^-^ heme reduction steps for MPO Compounds I and II in the presence of MLT. The MPO sample that was incubated with 100 μM MLT and treated with 400 μM H_2_O_2_ (total) led to the production of a MLT metabolite with elution time of 3.57 min, which was similar to elution time of AFMK alone (Fig. 4D). Consistent with these studies, Ximenes et al. similarly have observed two MLT metabolites (hydroxyl melatonin and AFMK) when MLT was incubated with MPO-H_2_O_2_ system or with stimulated neutrophils [52]. Thus, MLT prevents MPO heme destruction either by directly scavenging HOCl and/or inhibiting MPO chlorinating activity.

## Discussion

In this work, we show that MLT largely prevents MPO catalytic inhibition, attributed to MPO heme destruction and the generation of free iron associated with HOCl synthesis through its function as a potent MPO inhibitor and/or a HOCl scavenger. Thus, MLT may contribute to the reduction of the inflammatory process not only by inhibiting MPO and consuming HOCl, but also by diminishing the release and accumulation of free iron.

Recently, we have characterized an irreversible inhibition that is related to MPO heme destruction and the generation of free iron, when appropriate concentrations of self-generated HOCl are reached in the enzyme milieu [34]. These findings were recently confirmed by Paumann-Page et al [53]. The accumulation of the released HOCl in the solution mixture permits the competition with H_2_O_2_ on the catalytic site of MPO, which is in this case is the heme prosthetic group [34]. Hypochlorous acid interacts with both MPO-Fe(III) and Compound I and accelerates their conversion to Compound II [34, 54], or forms a relatively stable MPO-Fe(III)-OCl complex, which also converts to Compound II prior to heme destruction [34]. Compound II is a long-lived intermediate, and thus would be notably susceptible to HOCl assault leading to heme destruction [32]. In the absence of an MPO inhibitor, HOCl scavenger, or both, the degree of MPO heme destruction is significantly high in that only a small portion of the total enzyme (5–10%) is estimated to remain active after multiple cycles of HOCl synthesis [21]. Our current results demonstrate that the degree of MPO heme pocket alterations (e.g., by changes in the hydrogen bonds) mediated by MLT is not only sufficient to affect the interaction of Compound I with Cl^-^ preventing the generation of HOCl, but also prevents HOCl access to the heme moiety; thereby avoiding HOCl-dependent heme destruction. These results are consistent with our previous detailed kinetic studies, which showed the ability of MLT to inhibit MPO chlorinating activity despite the presence of high concentrations of Cl^-^ [17]. Melatonin competes with Cl^-^ and switches the MPO catalytic activity from a 2e^-^ oxidation to a 1e^-^ oxidation pathway. Under these conditions, MPO did not generate HOCl but still consumed H_2_O_2_ at slower rates. H_2_O_2_-selective measurements showed that MLT presence inhibits MPO peroxidase activity. This observation appears relevant even in the presence of alternative substrates because peroxidases like MPO are not saturated under physiological conditions [55]. These findings were also supported by theoretical modeling, which showed that indole compounds could be accommodated in the narrow regions of the active site pockets of MPO when the indole ring was situated parallel to the heme plane and close enough to the D pyrrole ring. Under the circumstances the side chain of the indole compound was directed toward the outside of the distal cavity [56].

It is clear from the MLT presence that the MPO chlorinating activity, but not H_2_O_2_, is implicated in MPO heme destruction and free iron release. This conclusion is consistent with previous studies by Paumann-Page et al. who showed that the MPO inactivation mediated by H_2_O_2_ is unlikely to take place in the presence of reducing substrates (100 mM Cl^-^) and under conditions in which the concentration of H_2_O_2_ does not accumulate [57]. The amount of MLT used (100 μM) in the current work is sufficient to inhibit MPO. Studies on the effect of MLT on HOCl production by neutrophils and purified MPO have showed that the concentration of MLT that inhibited HOCl production by 50% (IC_50_) was estimated to be 18 μM and reduced to 4 μM when superoxide was removed by addition of superoxide dismutase [52]. In contrast, the IC_50_ value, calculated from the initial rate of H_2_O_2_ consumption as a function of the MLT concentration was 3 μM [17]. Our HPLC analysis showed that the protection of MPO heme destruction mediated by self-generated HOCl occurred at the expense of MLT oxidation, which depends on the concentration of H_2_O_2_ used. Ximenes et al. showed the elution of two MLT metabolites when MLT was exposed to neutrophils [52]. In their system, the major and minor peaks were AFMK and a hydroxlyated melatonin metabolite, respectively. We similarly observed two peaks in our system; however, we believe the major peak was the hydroxylated intermediate when a lower concentration of H_2_O_2_ (1:2, MLT: H_2_O_2_) was used. In contrast, AFMK predominated when MPO was exposed to higher concentrations of H_2_O_2_ (1:4, MLT: H_2_O_2_). This alteration in the peroxidation turnover resulted in the reversal of the populations of the two MLT metabolites. Thus, prevention of MPO heme destruction depends on multiple factors including the bioavailability of HOCl, the presence of a capable 1e^-^ substrate that can compete with Cl^-^ switching the reaction from a 2e^-^ to a 1e^-^ oxidation pathway (e.g. ascorbic acid, superoxide, and nitric oxide), and the presence of HOCl scavengers.

Melatonin prevention of HOCl-mediated heme destruction is not limited to MPO, but also applies to other hemoprotein model compounds, such as hemoglobin, lactoperoxidase, catalase, as well as isolated human red blood cells [32–34, 52, 58, 59]. Earlier kinetic measurements have indicated that HOCl initially mediates the sequential formation of ferryl peroxidase-like intermediates, compounds I and II, followed by heme degradation [33, 34, 54, 60]. Hypochlorous acid can also mediate tetrapyrrole ring destruction independent of the iron molecule that resides in the porphyrin center [32]. A general chemical mechanism that describes the tetrapyrrole ring destruction resulting from the direct attack of HOCl and generation of multiple heme degradation products is well documented [20, 21, 32, 33, 37]. Because of MLT's ability to inhibit MPO, destabilize the Compound II intermediate, and/or directly scavenge HOCl, MLT could be considered an ideal component for prevention against HOCl mediated oxidative damage.

Although experiments that utilized methionine or taurine as a scavenger of HOCl showed that they could prevent HOCl-mediated MPO heme destruction [18] similar to MLT, there are important differences in the fundamental aspects. Melatonin and its precursors, unlike other HOCl scavengers, display a high affinity towards transition metal binding (e.g. iron (III), copper and zinc), and subsequently reduce their cytoplasmic availability [61–63]. In addition, several in vivo studies have shown that administration of MLT directly or indirectly neutralizes a variety of ROS, resulting in the reduction of lipid peroxidation, protein oxidation, and DNA damage, thus helping the immune system [11, 62–65]. One other factor that distinguishes MLT from other HOCl scavengers (e.g. taurine, cystine, cysteine and uric acid) is that its oxidation products have no biologically harmful sequelae [18, 66]. Melatonin reacts with HOCl to produce 2-hydroxymelatonin [47] at a rate sufficient to protect catalase against inactivation by this molecule [67]. Melatonin’s presence during MPO catalysis is associated with a significant diminution of free iron release, decrease in the intensity of the fluorescent heme degradation products, and reduction in different profiles of protein aggregation [47]. In contrast, taurine reacts with HOCl to form a less active oxidant taurine chloramine. It is important, however, to note that while chloramines are less reactive than HOCl, they can still oxidize thiols, thioethers and heme proteins, and thus extend the reactivity of HOCl [66, 68, 69].

The association between enhanced MPO expression and increased levels of free iron is characteristic of many inflammatory disorders including cardiovascular diseases such atherosclerosis, pulmonary diseases such as cystic fibrosis, neurodegenerative diseases such as Alzheimer’s disease as well as arthritis, diabetes, and has been found to be a risk factor for various cancers [28, 29, 31, 70–75]. As free iron accumulates, it disturbs body processes by replacing certain vital minerals such as zinc, copper, and manganese in many enzymes, depleting vitamins such as vitamin E and D, and may lead to chronic infection and inflammation [76]. Due to its properties as an excellent oxygen transporter, iron tends to stimulate the growth of tumor cells and bacteria [77–79]. Therefore, blocking the MPO chlorination machinery (MLT, tryptophan, and tryptophan analogs) [17, 35, 36] or scavenging HOCl (MLT, methionine, lycopine, taurine, and glutathione) might be a useful therapeutic approach in reducing free iron release in a wide variety of inflammatory conditions.
